# CLO: The cell line ontology

**DOI:** 10.1186/2041-1480-5-37

**Published:** 2014-08-13

**Authors:** Sirarat Sarntivijai, Yu Lin, Zuoshuang Xiang, Terrence F Meehan, Alexander D Diehl, Uma D Vempati, Stephan C Schürer, Chao Pang, James Malone, Helen Parkinson, Yue Liu, Terue Takatsuki, Kaoru Saijo, Hiroshi Masuya, Yukio Nakamura, Matthew H Brush, Melissa A Haendel, Jie Zheng, Christian J Stoeckert, Bjoern Peters, Christopher J Mungall, Thomas E Carey, David J States, Brian D Athey, Yongqun He

**Affiliations:** 1 US Food and Drug Administration, Silver Spring, MD, USA; 2 University of Michigan, Ann Arbor, MI, USA; 3 European Molecular Biology Laboratory, (EMBL-EBI), Hinxton, UK; 4 University at Buffalo, Buffalo, NY, USA; 5 University of Miami, Miami, FL, USA; 6 University Medical Center Groningen, Groningen, Netherlands; 7 RIKEN BioResource Center, Tsukuba, Japan; 8 Oregon Health & Science University, Portland, USA; 9 University of Pennsylvania, Philadelphia, USA; 10 La Jolla Institute for Allergy & Immunology, La Jolla, CA, USA; 11 Lawrence Berkeley National Laboratory, Berkeley, CA, USA; 12 OncProTech LLC, Ann Arbor, MI, USA

**Keywords:** Cell line, Cell line cell, Immortal cell line cell, Mortal cell line cell, Cell line cell culturing, Anatomy

## Abstract

**Background:**

Cell lines have been widely used in biomedical research. The community-based Cell Line Ontology (CLO) is a member of the OBO Foundry library that covers the domain of cell lines. Since its publication two years ago, significant updates have been made, including new groups joining the CLO consortium, new cell line cells, upper level alignment with the Cell Ontology (CL) and the Ontology for Biomedical Investigation, and logical extensions.

**Construction and content:**

Collaboration among the CLO, CL, and OBI has established consensus definitions of cell line-specific terms such as ‘cell line’, ‘cell line cell’, ‘cell line culturing’, and ‘mortal’ vs. ‘immortal cell line cell’. A cell line is a genetically stable cultured cell population that contains individual cell line cells. The hierarchical structure of the CLO is built based on the hierarchy of the *in vivo* cell types defined in CL and tissue types (from which cell line cells are derived) defined in the UBERON cross-species anatomy ontology. The new hierarchical structure makes it easier to browse, query, and perform automated classification. We have recently added classes representing more than 2,000 cell line cells from the RIKEN BRC Cell Bank to CLO. Overall, the CLO now contains ~38,000 classes of specific cell line cells derived from over 200 *in vivo* cell types from various organisms.

**Utility and discussion:**

The CLO has been applied to different biomedical research studies. Example case studies include annotation and analysis of EBI ArrayExpress data, bioassays, and host-vaccine/pathogen interaction. CLO’s utility goes beyond a catalogue of cell line types. The alignment of the CLO with related ontologies combined with the use of ontological reasoners will support sophisticated inferencing to advance translational informatics development.

## Background

Cell culturing dates back to as early as 1911 when Alexis Carrel attempted to grow living cells outside an organism. The establishment of the first human cell line, HeLa, in 1951 has since brought the fruitful development of other cell lines from different organisms. Cell lines have been commonly used in many aspects of biomedical research and experimentation. Mass production of cell line culture of animal cells is fundamental to the manufacture of viral vaccines and other products in biotechnology such as enzymes, synthetic hormones, anti-cancer agents, and immunobiologicals (e.g., monoclonal antibodies, interleukins, and lymphokines). However, it has been realized that cell lines are often contaminated by other lines – for example, the robust HeLa line has been shown to have widely contaminated many cell lines [1-3].

In addition to the cross-contamination, other issues exist in the domain of cell line representation. Due in large part to a history of bottom-up naming practices, cell line nomenclature has not been standardized or controlled by any centralized authority. This has made management and tracking of cell line information a difficult task, despite the existence of various public repositories and indexed catalogues available for open access. Moreover, cell line related terms are loosely interchangeable and inconsistently used across communities, such that terms like ‘primary cells’ , ‘primary cell culture’ , and ‘cell line’ have become loaded with conflated and ambiguous meaning. Confusion can also come from variability in how cell lines are categorized. This results in part from the wide range of methods for generating and modifying cell lines confer diverse attributes used in their classification. As we move toward the establishment of a centralized resource for cell lines, the ambiguity of cell line-associated terms needs to be clarified.

Many of the challenges can be addressed by the development of an ontology for cell lines, wherein the various cell line attributes can be normalized and based on agreement between users in the community. The different aspects of describing a cell line can be modularized by their corresponding source organism and anatomical part, modifications, and culturing methods, or related diseases.

The Cell Line Ontology (CLO) is a community-based ontology that covers the biological cell line domain. The CLO was originally presented in the International Conference on Biomedical Ontology (ICBO) in 2011 [4]. The original CLO was developed cooperatively by ontology editors from the University of Michigan Cell Line Knowledge base (CLKB) team, the EBI Functional Genomics Production Team, Cell Ontology (CL) [5] team, and the Bioassay Ontology (BAO) development team at the University of Miami. Subsequently, the Cell Bank of RIKEN BioResource Center (BRC) in Japan, the Ontology for Biomedical Investigation [6], and Reagent Ontology [7] joined the CLO development consortium. The CLO Consortium aims to unify publicly available cell line data from multiple sources to a standardized format based on a consensus design pattern derived from the establishment of CLO. This manuscript focuses on introducing recent updates on the CLO development.

## Construction and content

### CLO statistics

The development of CLO follows the OBO Foundry principles, including openness, collaboration, and use of a common shared syntax [8]. As a result, the CLO developers have been working to establish a common understanding and agreement of key CLO-specific terms. The CLO terms and definitions have been created and refined with the input from participants who utilize the CLO in their studies. In addition, the CLO also makes use of existing ontologies via an OntoFox import strategy [9]. The CLO is aligned with the Basic Formal Ontology (BFO; http://www.ifomis.org/bfo) version 2 (Graz release) by importing of all the class terms of BFO as its upper level ontology. Note that the preparation for BFO version 2.0 alignment has also been implemented in the event that its use becomes an OBO recommendation. Object properties imported from the Relation Ontology (RO) are used to represent relations in the CLO. Some terms from Spatial Ontology (BSPO), SemanticScience Integrated Ontology [10] and Information Artifact Ontology (IAO) [11] are also imported as part of the top-level ontology manager. Table 1 summarizes classes imported for the upper-level ontology operations, along with the classes utilized from other external ontologies to establish data integration and to support automated reasoning.

**Table 1:** **Summary of ontology terms in CLO and major source ontologies used in CLO as of November 21**^**st**^**, 2013**

**Ontology**	**Classes**	**Object properties**	**Annotation properties**	***Total***
CLO (Cell Line Ontology) specific	38453	2	0	38455
***Imported upper-level ontologies***				
BFO (Basic Formal Ontology)	22	38	0	60
RO (Relation Ontology)	0	85	1	86
BSPO (Spatial Ontology)	0	18	0	18
SIO (SemanticScience Integrated Ontology)	0	3	0	3
IAO (Information Artifact Ontology)	17	2	17	36
***Imported entities from other external ontologies***				
OBI (Ontology for Biomedical Investigation)	20	6	2	28
EFO (Experimental Factor Ontology)	149	1	1	151
CL (Cell Ontology)	269	0	0	269
UBERON	1315	0	0	1315
NCBITaxon (NCBI Taxonomy)	354	0	0	354
PATO (Phenotypic Quality Ontology)	22	0	0	22
GO (Gene Ontology)	299	0	0	299
PR (Protein Ontology)	5	0	0	5
DOID (Human Disease Ontology)	741	0	0	741
ChEBI (Chemical Entities of Biological Interest)	32	0	0	32
***Total***	41698	155	21	41874

The detail of total number of terms and the most recent update can be viewed at http://www.ontobee.org/ontostat.php?ontology=CLO.

In summary, CLO consists of over 38,000 terms for various cell line cells. These are mostly cell line information obtained from cell line records deposited at four cell line resources: the ATCC and HyperCLDB cell lines stored in the CLKB from the University of Michigan, Corriell Cell Lines processed by EBI, and the cell lines from the Cell Bank of RIKEN BioResource Center (BRC). Almost 300 cell line entry descriptors of cell types were imported from CL and over 1,300 anatomical entities were imported from UBERON [12]. CLO currently contains information of cell lines derived from more than 350 species (NCBITaxon entities). Biomedical experiment related terms were imported from OBI and EFO [13]. When applicable, components from the following resources: Gene Ontology (GO), Phenotypic Quality Ontology (PATO), Protein Ontology (PRO) [14], Chemical Entity of Biological Interest (ChEBI), and Human Disease Ontology (DOID) are imported into CLO based on available information in the cell line cell records.

The CLO was developed using the format of W3C standard Web Ontology Language (OWL2) (http://www.w3.org/TR/owl-guide/). The latest CLO is available for public view and download at http://code.google.com/p/clo-ontology/. The latest version of the CLO is also available for visualization and downloading from Ontobee (http://www.ontobee.org/browser/index.php?o=CLO) or NCBO’s BioPortal: (http://purl.bioontology.org/ontology/CLO). The source code of CLO is open and freely available under the Apache License 2.0.

### Alignment of core domain concepts between CLO, OBI, and CL

As part of the CLO refactoring process, a working group was established between members of several key open biomedical ontologies where cell line-related entities are represented, including the Cell Ontology (CL) and the Ontology for Biomedical Investigation [6]. The goal of this group was to align modelling related to cultured cells in accordance with OBO Foundry principles of orthogonality and re-use. One key outcome of this work was the integration of inconsistent representations into a single shared model. Classes representing key concepts were implemented in the CL, CLO, and OBI - with the CL as a home for high-level *in vitro* cell modelling (e.g. *cultured cell*), the CLO as a home for more specific *cell line cell* and *cell line* classes, and the OBI as a home for experimental entities related to these cell lines (e.g. *cell line culture* and *establishing cell line* classes). As a result, each term has a single representation that is re-used between ontologies through established import mechanisms. A second key outcome of this alignment work was the crafting of clear consensus definitions for common but ambiguous domain terminology, including a careful characterization of the term 'cell line' itself. Updated definitions for a selection of key CLO classes resulting from this work are detailed below.

A *cell line* is defined as a genetically stable and homogenous population of cultured cells that shares a common propagation history (i.e. has been successively passaged together in culture). This view clarifies two key confusions surrounding the term ‘cell line’. The first relates to the scale at which the term applies, here referring to discrete experimental populations rather than maximal collections representing an entire lineage (e.g. the collection of all HeLa cells that exist). The second concerns the criteria that establish when a cultured cell population qualifies as a ‘line’. By applying ‘cell line’ to experimental populations with a shared culture history, we define the term consistently with its most prevalent usage in domain discourse, and in a way that is most fit for data annotation needs, as it represents populations that are actually cultured, experimented upon, and shared in the practice of science.

**Figure 1:**
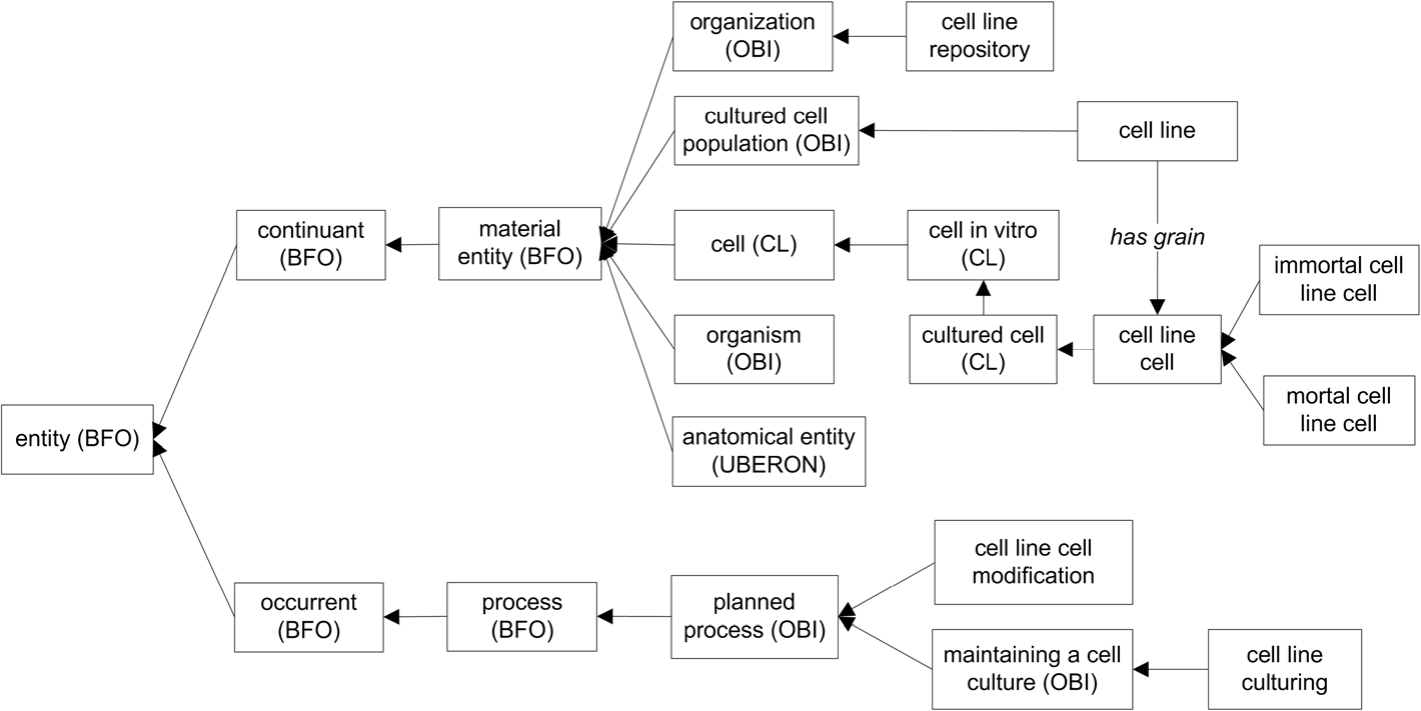
**The top level CLO hierarchical structure and key ontology terms.** Terms imported from other ontologies are indicated by the ontology abbreviations inside parentheses. Terms without an identified source are CLO terms. All the arrows indicate the ‘is_a’ relation except the explicitly labelled ‘has grain’ relation.

A *cell line cell* is defined simply as a cultured cell that is part of a cell line. This class is a child of *CL:cultured cell*, defined as a cell *in vitro* that is or has been cultured *in vitro* (Figure 1). Cell line cells, like cell lines, can exist in active culture or stored in quiescence. The representation of each specific cell line (e.g. HeLa, HEK293) is implemented at the scale of individual cells, such that ‘cell line cell’ is the root of the core CLO hierarchy of cell line types.

A *clonal cell line* is defined as a cell line that derives from a single cell that is expanded in culture. Feedback from community experts and stakeholders initiated the representation of this specific type of cell line as a key experimental resource with unique and valuable attributes.

Finally, a *cell line culture* represents an actual physical culture of cell line cells that is an input to experimental processes, and is comprised cell line cells and the media along with any added components. This term is intended to cover actively propagated cultures as well as those kept frozen.

Through the alignment efforts summarized above, we have increased the utility of the CLO as a community resource for standardizing reference to domain concepts and facilitating the exchange and discovery of cell line related information.

### Basic CLO cell line design pattern

**Figure 2:**
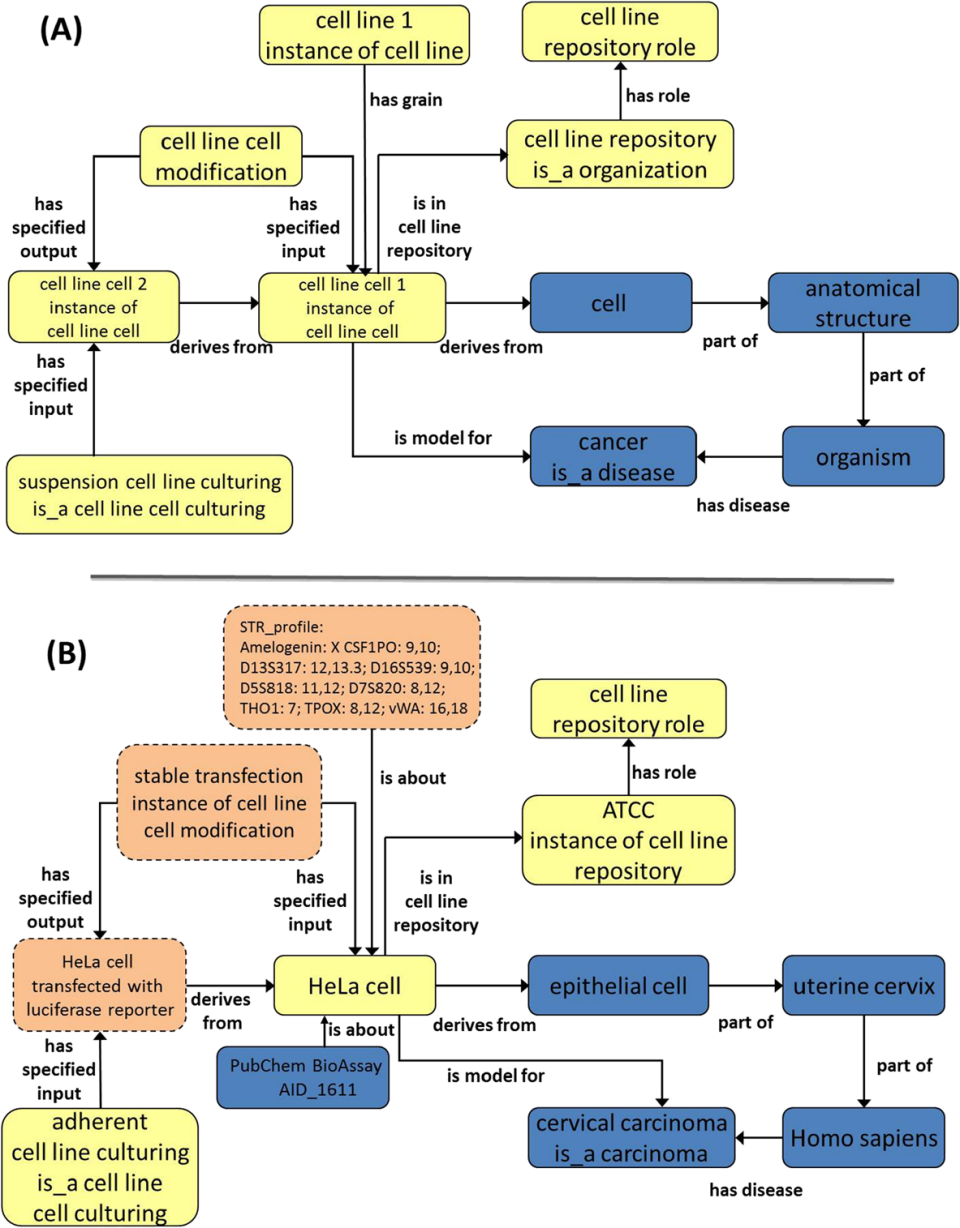
**CLO cell line design pattern and an example. (A)** Generic CLO design pattern. Terms imported from external ontologies are displayed in blue boxes, while CLO-specific terms are displayed in yellow boxes. **(B)** An example of HeLa cell representation by applying CLO design pattern with extended information obtained from the BAO development (shown in dashed orange boxes) with details of culturing method, cell line modification, and STR profiling. Boxes represent instances, some labelled by the class they are an instance of. The relations used to link the boxes are instance-level relations. In several cases the parent class is also noted in the box using an is_a relation.

The basic CLO design pattern represents organ anatomy, cell types, disease and pathology, source information in the form of ownership and derivation where cell lines are related to each other, and technical information such as culture conditions (Figure 2A). A core design pattern implemented for, cell line cell, describes derivation from an *in vivo* cell, which is in turn, part of some anatomical structure, and in turn, anatomical structure is part of a species of organism. In addition, diseases borne by the source organism can be captured in this pattern when known. An object property ‘is model for’ is defined to represent a relation between a cell line cell and a disease where the cell line cell is a model system for studying the disease. An example of applying this general design pattern to a concrete example is shown in Figure 2B where the design pattern is used to represent a HeLa cell transfected with luciferase reporter in BAO. In this example, a HeLa cell transfected with a luciferase reporter ‘derives from’ HeLa cell through a stable transfection that ‘is a’ cell line cell modification process. The transfected HeLa cells are part of or specified input of cell line culturing process, specifically, adherent cell line culturing process. This cell line cell also inherits the pathological cell properties from *HeLa cell* (as a cell line cell), which ‘derives from’ some *epithelial cell* that is ‘part of’ some *uterine cervix*, which is ‘part of’ an organism *Homo sapiens.* This specific instance of Homo sapiens also ‘has disease’ of some *cervical carcinoma,* which ‘is a’ *carcinoma* and *HeLa cell* is a cell line model for studying this cancer. Relations of knowledge in multiple domains are shown in this example of a cell line cell annotation in BAO with the semantic infrastructure provided by CLO. Modification of a cell line cell can give rise to another derived cell line cell, which is also described by ’derives from’ relation. A cell line cell is specified input of some special culturing process (*CLO:cell line cell culturing*, a subclasss of *OBI:maintaining a cell line culture*), which can differ from culturing one cell line cell to another (*e.g.*, *suspension cell line culturing* or *adherent cell line culturing*). The relation ‘derives from’ is not a transitive relation. The ‘derives from’ relation could be transitive if, and only if, there are no confounding environmental and/or experimental conditions that affect the cell line’s characteristics (such as cross contamination). The cell line cell culturing reflects a particular culturing condition or growth mode (*e.g.* suspension or cell surface adhesion). A cell line is supplied, maintained, or catalogued by a specific organization such as American Type Cell Culture (ATCC) that ‘has cell line repository role’. Since relation terms such as ‘supply, ‘own’, or ‘manage’ have not been fully developed in any ontology, we have created a CLO-specific relation reflecting this activity with the label ‘is in cell line repository’. This object property designates the representation of a particular cell line’s information in such repository*.* This is an update from the previous version that utilized relation ‘mentions’ to describe this activity. The relation of class label ‘mentions’ was used in CLO’s previous release to cover all general references to a source, such as MeSH, as CLO made use of MeSH entries that neither supplied, owned, nor catalogued, but rather just listed (or mentioned) cell lines instead. In this version of CLO, MeSH reference was established via ‘seeAlso’ annotation property value.

The basic design model can be extended in different aspects. In addition to having an organism acting as a ‘bearer of’ a cancer, or a disease, the Coriell cell line repository has often provided the information that associates a cell line to a specific disease using the relation ‘is model for’. With this relation, there is no need to connect a cell line to a disease through the organism. One drawback of directly applying this design pattern is that the cell line layout in the original CLO structure was mostly one-layer with one level of subclassing where majority of the cell line cell classes are all immediate children of the parent class *‘mortal’* or ‘*immortal*’ cell line cell. Logical reasoning is the primary approach to infer a possible hierarchy. However, such inferential reasoning of this one-level subclassing information approach is not optimal as the scalability of computational resources and power can be problematic in processing a large ontology. Another possibility to ease the situation is to introduce a pre-composed differentia criteria assertion for classification. However, if we consider pre-composing all possible differentia parents in CLO (e.g. by organism, by cell type, by anatomical entity, or by culturing methods), construction of an asserted hierarchy based on all criteria will lead to scalability and maintenance issues and, we would face the situation of exponential growth of the pre-composed differentia parent hierarchy and risks the generation of logical, but biologically nonsensical class creation. A proposal for the solution to overcome this challenge has been addressed in the updated version as outlined below.

### Restructuring of cell line cell hierarchy

There are approximately 38,000 immortal cell line cells in the CLO. To list them all as a single layer under *immortal cell line cell* is problematic. For example, a one-layer structure would miss a classification that may be useful to have as intermediate layer terms (e.g., *immortal epithelial cell*), which have been frequently used. These intermediate layer terms need to be added to the CLO, and specific cell lines can be asserted under these terms to support direct usage. After intermediate terms are added, specific cell line cell terms can be asserted to under these intermediate terms, supporting effective query and data analysis. The accurately asserted hierarchy can also prevent time consuming reasoning process given the large number of cell line cells in the CLO.

**Figure 3:**
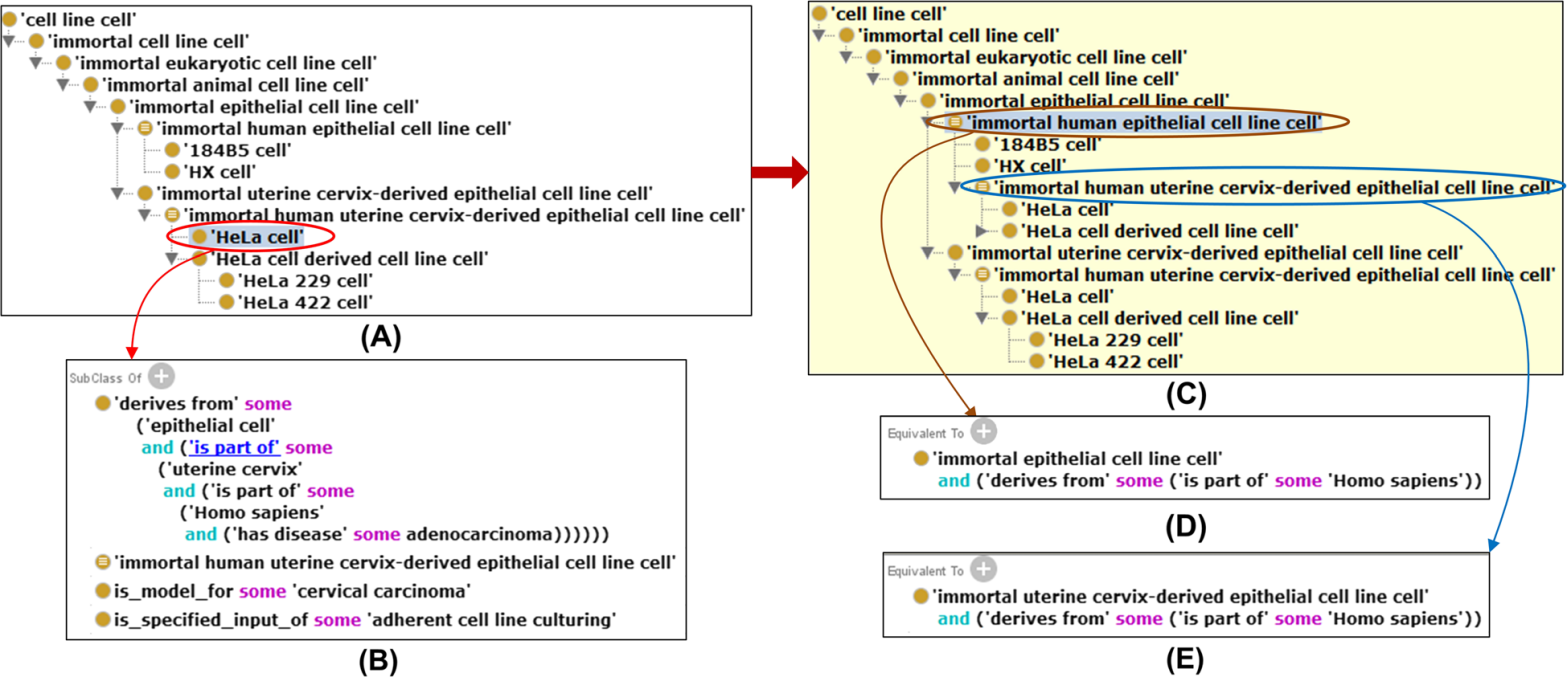
**Demonstration of restructured CLO cell line cell hierarchy and the application of reasoning. (A)** asserted HeLa cell hierarchy. Five intermediate terms between ‘immortal cell line cell’ and ‘HeLa cell’ have been generated in CLO. **(B)** The SubClass axioms of ‘HeLa cell’. **(C)** Part of an inferred CLO hierarchy. Compared to the asserted hierarchy in Figure 3A, the term ‘immortal human uterine cervix-derived epithelial cell line cell’ has been inferred as a subclass of ‘immortal human epithelial cell line cell’. **(D)** The equivalent class definition for ‘immortal human epithelial cell line cell’. **(E)** The equivalent class definition for ‘immortal human uterine cervix-derived epithelial cell line cell’. The OntoFox tool [9] was used to generate a subset of CLO that includes HeLa cell-related terms. The screenshots were generated using the Protégé OWL editor. The ELK version 0.3.2 ontology reasoner was used for reasoning.

To solve these issues, the updated CLO includes a new multi-layer hierarchical structure based on well-defined design patterns, making it easier to browse, query, and perform automated classification. The hierarchical structure of cell line cells in the CLO is built up primarily based on three criteria: *in vivo* cell type, *in vivo* tissue types, and organism species, from which cell line cells are derived. Currently all cell line cells in the CLO are immortal, so the term ‘immortal’ is used in all the intermediate cell line cell terms. We first used specific cell types defined in the CL to generate cell type specific cell line cells, e.g., *immortal fibroblast cell line cell*. The CLO contains 271 cell type terms imported from the CL (Table 1). New CLO terms defined based on CL cell types follow the same hierarchical structure of corresponding CL terms. Secondly, we added an anatomical region (e.g., liver), defined in UBERON, to the specific cell line to generate an organ-specific term, i.e., immortal liver-derived fibroblast cell line cell. CLO currently contains over 1,300 anatomical structure terms imported from UBERON (Table 1) and used for this process. Lastly, the organism species (e.g., Homo sapiens defined by NCBITaxon) was used to generate an organism-specific intermediate layer, e.g., immortal human liver-derived fibroblast cell line cell. The CLO includes cell line cells originated from over 100 different animal species. In total, we have generated over 1,000 intermediate classes in CLO.To better illustrate the restructuring strategy, the HeLa-related hierarchy is used as an example (Figure 3). Five intermediate terms were generated between ‘HeLa cell’ and ‘immortal cell line cell’ (Figure 3A). To automatically generate these intermediate layers, three Java programs were developed internally to use the following axiom defined for the HeLa cell (Figure 3B):

'derives from' some

('epithelial cell'

and ('is part of' some ('uterine cervix'

*and ('is part of' some 'Homo sapiens'))))*The ‘epithelial cell’ (CL_0000066) defined in the CL includes parent terms ‘animal cell’, ‘eukaryotic cell’, and then ‘native cell’. Based on this hierarchy, the CLO includes the first three layers of superclasses of ‘HeLa cell’ under ‘immortal cell line cell’ (Figure 3A). Based on the UBERON ‘uterine cervix’ linkage, a new term ‘immortal uterine cervix-derived epithelial cell line cell’ is generated. Lastly, the term ‘immortal human uterine cervix-derived epithelial cell line cell’ is generated as a new immediate superclass of ‘HeLa cell’ (Figure 3A). It is noted that the CLO includes a new term called ‘immortal human epithelial cell line cell’ that is not an asserted superclass of the ‘immortal uterine cervix-derived epithelial cell line cell’ (Figure 3A). However, a reasoning process infers such an is_a relation (Figure 3C), based on the related equivalent class definitions (Figure 3D and E).

### Adding new cell line cells in Japan RIKEN Cell Bank to CLO

RIKEN cell lines are associated with unique IDs managed in the BioResource Web Catalog (http://www.brc.riken.jp/lab/cell/english/search.shtml). When integrating these cell lines into CLO, they are merged and assigned new CLO IDs. First, an Excel worksheet with a fixed template was generated to represent 1622 cell lines from the Riken Cell Bank. It is noted that these cell lines do not include any stem cells stored in the RIKEN Cell Bank. The data prepared in the worksheet was used as input for the Ontorat tool to generate an OWL file that can be directly imported and merged to CLO. The Ontorat program (http://ontorat.hegroup.org/) [15] acts by following ontology design patterns. Specifically, the immediate parent terms of individual RIKEN cell line cells were manually identified based on the reconstructed cell line cell hierarchy described above, and inserted into the Excel worksheet. A new CLO 'label' was generated by combining a unique RIKEN cell line number (e.g., RCB0871) with the word 'cell', for example, 'RCB0871 cell'. The RIKEN cell line names are defined as alternative terms. Other information such as cell line originators and registers are also included in the CLO annotations for these new RIKEN cell lines.

## Utility and discussion

### CLO applications

**Figure 4:**
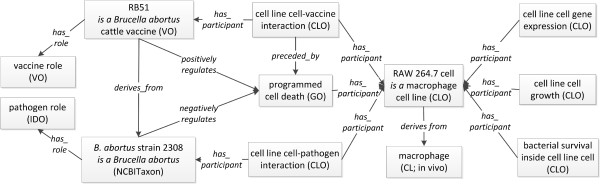
**CLO application in modelling the cell line cell-vaccine/pathogen interactions.** As reported in a reference [16], the live attenuated *B. abortus* cattle vaccine strain RB51 positively regulated programmed cell death of RAW264.7 cell, a macrophage cell line cell. However, its wild type virulent strain 2308 negatively regulated the programmed cell death. The infected cells underwent different cellular processes after the interaction with the vaccine or pathogen. All the boxes represent instances, labelled by the class they are an instance of.

Cell lines are routinely used in various biological and biomedical studies. For example, many cell lines have been used as models for analysis of *in vivo* host -pathogen interactions and vaccine-induced cellular immune responses. Brucellosis is one of the most common zoonotic infectious diseases in humans and other animals worldwide. Brucellosis is caused by virulent *Brucella*, an intracellular bacterium that replicates and survives inside macrophages. Virulent *Brucella* strains, including *B. abortus* strain 2308, survive inside macrophages and inhibit macrophage cell death for prolonged replication. However, the live attenuated *B. abortus* cattle vaccine strain RB51 triggers (positively regulates) programmed cell death of RAW264.7 cell, a macrophage cell line cell used for *in vivo* cell modelling [16]. Further study found that a cell death in the cell line cells is mediated by caspase-2. The infected cell line cells also undergo different cellular processes including gene expression changes, cell growth phenotypes, and bacterial replication [16,17] (Figure 4). The phenotypes shown in vitro using cell line cells could also be verified *in vivo* and using primary macrophages directly isolated from mouse [16].

The European Bioinformatics Institute (EBI) Functional Genomics Group has developed ArrayExpress and an ontology-based linked data system for direct processing and query of microarray data from different studies, including cell line cell studies. Given the usage of cell lines in microarray studies, the development of a comprehensive cell line cell ontology is needed to support for efficient query of functional genomics studies. EBI has been using an internally developed Coriell Cell Line Ontology for ArrayExpress microarray data analysis [18]. Since the contents of the Coriell Cell Line Ontology are now incorporated into CLO and the CLO structure and its alignment with existing ontologies have significantly improved and EBI will utilize the updated CLO for their future array data analysis. A clear CLO use case lies in the usability of the ArrayExpress database. Researchers often query cell lines with cross-reference information to the cell lines’ corresponding tissues and cell types. The reconstructed CLO intermediate term hierarchy provides an effective mechanism to support this type of query.

The Bioassay Ontology (BAO) describes bioassays and results obtained from small molecule perturbations, such as those in the PubChem database [19]. To describe and annotate cell-based PubChem assays and screening results comprehensively, the BAO is being extended through collaborative development of the CLO. By integrating the BAO with the CLO, those cell lines that are typically used in cellular assays are added into the CLO. Based on the demands of BAO bioassay modelling, extended parameters are being added to the CLO, including different sources of cell lines (normal/healthy tissue, pathological tissue, or tumor), cell modification methods (plasmid transfection, viral transduction, cell fusion, *etc.*), culture conditions (composition of culture medium), morphology (epithelial, lymphoblast, *etc.*), growth properties (adherent or suspension), short tandem repeat (STR) profiling and other properties that are relevant for cellular screening [19]. As a demonstration of the use of CLO in BAO bioassay modelling, the STR profiling analysis with the HeLa cell line has been modelled in the context of a PubChem assay (AID 1611) (Figure 2B). In the PubChem assay, HeLa cells were modified by stable transfection with a heat shock promoter driven-luciferase reporter gene construct. In this assay, the modified HeLa cells were used to screen for compounds that could induce heat shock transcriptional response as a potential therapeutic for Huntington’s disease and amyotrophic lateral sclerosis (ALS) (http://pubchem.ncbi.nlm.nih.gov/assay/assay.cgi?aid=1611).

As shown in the use cases above, integrating a formal representation of cell lines will benefit researchers in interpreting and analysing host-pathogen interactions and cell-based screening results, and better understanding the mechanisms of genotype-phenotype mappings. Formally described cell lines and related experimental conditions can help researchers better understand cellular and immunological pathways, and support rational design of novel assays, for example, with respect to choosing the best cellular model system, in identifying which modified cell lines are available and in demonstrating which ones work best in existing assays.

## Discussion and perspectives

The initial attempt to represent cell line information systematically in the CLKB has been performed by imposing ontological semantics and structure on the CLKB and transferring it to the CLO. The CLO now provides a better organization of cell line related information. This, in turn, aids further ontology application in modern translational bioinformatics, especially in the domain of concept mapping and alignment to represent knowledge in a complex biological system. As shown in this paper, contributions by the community collaborators have customized the CLO to accommodate the various use cases in the biomedical domain. The CLO aligns with existing OBO Foundry ontologies including CL, OBI, and UBERON. The governance of the CLO is achieved by importing upper-ontology classes from other OBO Foundry ontologies, namely, BFO, RO, OBI, and OGMS. Based on the alignment and new CLO design pattern, intermediate cell line cell terms were generated and new cell line cells from Japan RIKEN Cell Bank were included in the CLO. This paper also introduces many use cases utilizing CLO ontological features.

The recent development of the Cell Culture Ontology (CCONT) has resulted in an ontology with overlapping cell line information to CLO [20]. CCONT inherits a parent cell line definition from the Experimental Factor Ontology (EFO) [13] that describes a cell line as a population of cells cultured *in vitro*. This shifts the focus of cell line representation from biologically-defined individual cells to the experimental perspective of a cell line population culturing description. CCONT has not yet aligned with CL and OBI and intermediate terms of tissue and organism have not been implemented. CCONT, however, introduces many useful cell line-associated properties such as *safety classification* and *cytogenetics*. With the complementary components to describe a cell line related information shared between the CLO and CCONT, it is possible that future collaboration may be initiated for the benefit of the community.

The current primary focus of the CLO does not yet encompass the consideration of stem cell derived cell lines. A generally accepted definition of a stem cell line is a self-renewing population of cells with the ability to differentiate into multiple distinct cell types [21]. In this definition, human stem cells have their origin in a variety of cell types ranging from pluripotent cells derived from embryos (human embryonic stem cell – hESC) to adult stem cells derived from various fully developed tissues such as blood, or bone marrow. The ability to differentiate into distinct cell types is more limited in adult stem cells in comparison to the pluripotency in hESC. In addition to having multiple cell type origins, stem cells can also differentiate into multiple distinct cell types under different conditions. Therefore, defining stem cell-derived cell lines based on their origin tissue/cell where they are created (as mandated by CLO’s design pattern) may not be sufficient to yield the information that experimental researchers need. Compared to stem cells, regular immortal cell line cells have largely unchanged cell line property characteristics in the *passaging* process*.* The issues surrounding the correct ontological representation of stem cell line cells are currently under investigation at a community level.

Confusion created by mislabelling/misidentification due to cross contamination and naming ambiguity has led to the need for cell line authentication and management, especially when cell lines are used as disease models in drug-discovery screening projects. A few attempts to resolve the situation have been introduced to the community. The ATCC initiated the ATCC Standards Development Organization (ATCC SDO) in 2007, leading to new consensus standards to authenticate cell lines by short tandem repeat (STR) DNA profiling (http://www.atcc.org/~/media/PDFs/STR_Profiling.ashx). Although STR profiling provides an online service for human cell line authentication, there exists no unified cataloguing data source that lends itself as a structured information system with the ontological capability to manage the information, transfer knowledge, and assist in knowledge discovery. We expect that a future collaboration between the CLO , the ATCC and other partners will provide a more effective way to support such an effort.

The CLO will contribute to the wider dissemination of cell line information for improving access and promoting the common use of cell lines as biological resources. Recently, “Linked Open Data (LOD)”, a set of methodologies for exposing, sharing, and connecting pieces of data, information, and knowledge on the World Wide Web (WWW) using URI and Resource Description Framework (RDF) coding has been proposed as a strategy forming the collective intelligence by the integration of data throughout the entire Internet. The CLO is fully compatible with the LOD strategy and has the potential to promote wider dissemination, easier use in local data analyses, and the expansion of public data sources by enabling distributed (non-centralized) efforts by the user community for cell line data. In RIKEN, the RDF/OWL based integrated database (SciNetS: https://database.riken.jp/) [22] and open data archive (BioLOD: http://www.w3.org/wiki/HCLSIG/LODD/Data), will utililize CLO for the data format of cell lines. Furthermore, the coordinating efforts in CLO developments with other biomedical ontologies such as those in the OBO Foundry will promote broader integration of cell line information with other domains in life science.

## Conclusions

Capabilities embedded within the CLO’s structure facilitate knowledge transfer and discovery that a simple catalogue of cell line cells could not achieve. Automated reasoning and alignment with other related ontologies study will expand the network of knowledge much needed for the future translational informatics development. Not only will the ontology backbone of CLO assist in this development, but the uniform knowledge base of over 38,000 cell line cells in CLO also makes CLO a reference resource for translational informatics. Ambiguity introduced by mislabelling of cell lines from various factors can also be minimized by using the CLO as a reference. A uniform representation of each cell line’s properties along with the curation of verifying information from other knowledge sources will minimize errors in experimental reporting.

The CLO Consortium initiative is the first collaborative attempt among our partner institutes as listed here to facilitate cell line data discovery and knowledge transfer to aid integrative translational biomedical research. As the CLO is an on-going community-driven project, it will continue to grow and evolve to overcome challenges that surface in the translational domain. We encourage all parties to participate by contributing their domain knowledge and expertise in this collaborative movement.

### Availability

Ontology: http://purl.obolibrary.org/obo/clo.owl

Homepage: http://www.clo-ontology.org/

## Abbreviations

BAO: BioAssay ontology; BFO: Basic formal ontology; BRC: Riken Bioresource Cell Bank; CCONT: Cell culture ontology; ChEBI: Chemical entities of biological interest; CL: Cell ontology; CLKB: Cell line knowledgebase; CLO: Cell line ontology; DOID: Human disease ontology; EBI: European Molecular Laboratory (EMBL) European Bioinformatics Institute; EFO: Experimental factor ontology; GO: Gene ontology; ICBO: International Conference on Biomedical Ontology; IDO: Infectious disease ontology; NCBITaxon: NCBI Organismal Classification; OBI: Ontology for Biomedical Investigations; OBO Foundry: Open Biology Ontology Foundry; OGMS: Ontology for General Medical Science; PR: Protein ontology; PATO: Phenotypic quality ontology; ReO: Reagent ontology; RO: Relation ontology; VO: Vaccine ontology.

## Competing interests

The authors declare no competing interests.

## Authors’ contributions

SS takes the role of the primary developer and has led CLO project from its initial state in CLKB to the current development. ZX and YLiu developed software programs and supported ontology updates. YLin is a supporting team member in CLO development. TFM and ADD provided CL support and ensured the alignment between the CL and the CLO. UDV and SCS helped develop the CLO design pattern and provided a case study in the application of CLO in BAO. CP, JM, HP led the effort of aligning CLO to the Coriell Cell Repositories’ cell line data organization, contributed to the CLO design pattern and provided the Coriell cell line dataset in OWL format. TT, KS, HM, and YN provided the Riken BRC cell line dataset and worked with YH to transform the dataset to CLO. MHB and MAH provided a case study in the application of CL in ReO and supported the alignment of CLO and OBI. JZ, CJS, and BP contributed in the alignment of CLO to OBI. CJM provided consultation in UBERON usage. DJS was one of the lead developers of CLKB. TEC and BDA provided their input on the domain expertise of cell culture under laboratory environment and translational informatics, respectively. As another primary developer of CLO, YH designed the CLO hierarchy reconstruction algorithm and built the use case of cell line cell-vaccine/pathogen interactions. All authors read and approved the final manuscript.
